# Analysis of Reported Cases of *Giardia lamblia* and *Cryptosporidium* spp. Infection in Children from Aragón (Northeast Spain) During the Period (2012–2021)

**DOI:** 10.3390/microorganisms13020298

**Published:** 2025-01-29

**Authors:** Laura Lafarga-Molina, Encarnación Rubio, Cristina Seral, Antonio Rezusta, Pilar Egido Lizán, Carmen Malo Aznar, Josep-Oriol Casanovas-Marsal, María Teresa Fernández Rodrigo, Pilar Goñi

**Affiliations:** 1 Department of Physiatry and Nursing, Faculty of Health Sciences, University of Zaragoza, Domingo Miral Street s/n, 50009 Zaragoza, Spain; llafargam@salud.aragon.es; 2 Department of Microbiology, Paediatric, Radiology and Public Health, Faculty of Medicine, University of Zaragoza, Domingo Miral Street s/n, 50009 Zaragoza, Spain; erubio@unizar.es (E.R.); pgoni@unizar.es (P.G.); 3 Nursing Department, University Hospital Miguel Servet, 50009 Zaragoza, Spain; 4 Group of Water and Environmental Health, Institute of Environmental Sciences (IUCA), Domingo Miral Street s/n, 50009 Zaragoza, Spain; 5 Aragón Health Research Institute (IIS Aragón), University Clinic Hospital Lozano Blesa, University of Zaragoza, 50009 Zaragoza, Spain; cseral@salud.aragon.es; 6 Networked Biomedical Research Center for Infectious Diseases, CIBER INFECT, Carlos III Health Institute, 28029 Madrid, Spain; 7 Department of Microbiology, University Hospital Miguel Servet, Padre Arrupe Street s/n, 50009 Zaragoza, Spain; arezusta@salud.aragon.es (A.R.); pegido1@gmail.com (P.E.L.); 8 General Directorate of Public Health, Department of Health, Government of Aragón, 50017 Zaragoza, Spain; mcmaloa@aragon.es; 9 Aragón Health Research Institute (IIS Aragón), Juan Bosco Street 13, 50009 Zaragoza, Spain; jocasanovas@iisaragon.es; 10 Group SAPIENF, Domingo Miral Street s/n, 50009 Zaragoza, Spain

**Keywords:** parasitology, epidemiology, child, disease transmission, infectious

## Abstract

Giardiasis and cryptosporidiosis, caused by *Giardia lamblia* and *Cryptosporidium* spp., are parasitic infections transmitted through faecal–oral routes or contaminated water. Although less common in Spain compared to developing countries, they pose a public health concern, particularly for vulnerable groups like children and immunocompromised individuals. This study aims to analyse the cases reported to the Microbiological Information System (MIS) in children between 2012 and 2021, as well as their distribution across sociodemographic variables. Proportions and infectivity rates were determined for epidemiological and sociodemographic data, and the incidence rate for giardiasis and cryptosporidiosis was calculated annually and by health sector. The variables analysed included sex, age, health sector and weather. For both diseases, there was a significant decrease in the number of cases in 2020, suggesting the importance of person-to-person transmission. Children were infected by *Giardia* in significantly higher proportion (*p* < 0.001), being the majority in age groups 5–14 years, while the proportion of boys and girls infected by *Cryptosporidium* was almost identical (1.4% vs. 1.3%), in children aged 2–4 years. Periodically there was a significant increase in cases of cryptosporidiosis, apparently related to the presence of torrential rains. Transmission is related to increased temperature and rainfall. Person-to-person transmission in the paediatric population needs further investigation. This study provides the foundation for future research on the evolution of cases of giardiasis and cryptosporidiosis in Spanish children. The data emphasise the need for informational campaigns on hygienic measures and efforts by public health authorities to maintain water resources in optimal condition to prevent parasite spread.

## 1. Introduction

*Giardia lamblia* and *Cryptosporidium* spp. are parasitic protozoa that cause gastrointestinal diseases worldwide. In low-income countries, particularly in areas with poor hygiene and overcrowding [1,2,3], these parasites contribute to malnutrition in children, which can be severe [4]. Additionally, in developed countries, the presence of asymptomatic cases, coupled with the implementation of sanitation and water purification systems, has led to a false perception of eradication. This misconception contributes to the perception of these diseases as exotic, and they are evident in the occurrence of outbreaks such as those reported to the national surveillance systems in Denmark, Finland, and Norway between 1998 and 2011, which affected a total of 85,995 individuals, with between 4 and 18 outbreaks occurring each year due to water resources [4,5,6]. Waterborne transmission and person-to-person contact are the most common mechanisms of human infection, with the potential involvement of contaminated raw food ingestion, and contact with animals or insects [7,8]. In general, the most vulnerable population consists of children starting school, due to their immature immune systems [9] and frequent interactions with peers [10,11]. Therefore, the outbreaks in day-care centres are common [5,11,12].

The symptoms caused by infection vary, ranging from asymptomatic cases to acute or chronic conditions, and can lead to dehydration and malnutrition in situations of immunosuppression or specific circumstances [9,13]. In addition, nonspecific symptoms are common, making detection and diagnosis difficult [14]. In this context, the spread of *Giardia* clusters causing asymptomatic infections has been previously reported [15]. *Cryptosporidium* also causes outbreaks periodically, such as the one in the autumn of 2023, which affected 480 people in Zaragoza city (Aragón; Spain) [16], highlighting the presence of reservoirs within the population and the possibility that its diagnosis is underestimated.

Both giardiasis and cryptosporidiosis are classified as notifiable diseases [17]. For this reason, the European Centre for Disease Prevention and Control (ECDC) monitors and surveys the epidemiological situation of giardiasis and cryptosporidiosis in Europe, using data reported by European countries. In Spain, cases are recorded in the Microbiological Information System (MIS) of the National Epidemiological Surveillance Network (RENAVE) [17,18], which collects data from regional MIS.

This study was conducted in the Autonomous Community of Aragón, located in the northeast of Spain. In terms of healthcare, the region of Aragón is divided into eight health zones [19]. Each zone has reference hospitals, whose Microbiology and Parasitology diagnostic laboratories process data and export them to the information system [18]. According to legislation [20], the Autonomous Community of Aragón has its own MIS, through which it reports communicable diseases and epidemiological situations.

Aragón has consistently reported cases of giardiasis and cryptosporidiosis since 2012, being the Spanish region with the highest number of *Giardia lamblia* infection cases in 2012 (224 cases) [21]. In the case of cryptosporidiosis, the difference in the number of cases compared to other regions is not as large [21].

Interestingly, Aragón is one of the Spanish regions where a significant amount of data is reported, but according to a large literature review, no studies on the prevalence of these protozoa have been conducted in the region. The few studies carried out in Spain in recent years [22] focus on their presence in vegetables, companion animals, environmental samples, or diagnostic methods [23,24]. In addition, assemblages and genotypes transmitted among humans during the years 2007–2015 have been studied, but the prevalence of giardiasis and cryptosporidiosis has not been investigated [25,26]. Recently, the study of giardiasis and cryptosporidiosis in the paediatric population, especially in asymptomatic children, has gained significant attention, as it has been shown that this group is particularly vulnerable to parasitic infections due to their immunological status. In cases where no symptoms are present, the infection may lead to chronic conditions such as irritable bowel syndrome. Therefore, research into the early diagnosis and prevention of these diseases in children has critical importance [13,27]. Access to real data and their analysis are the main difficulties, due to the high prevalence of asymptomatic patients, reaching 17% in the case of giardiasis in children, and in relation to cryptosporidiosis, 1% in previous studies [27].

Given the prevalence of infections caused by both protozoa, it is essential to investigate their distribution over time in the Autonomous Community of Aragón, particularly their prevalence in the paediatric population and the main transmission routes, in order to establish preventive measures. This study aims to analyse the proportion of giardiasis and cryptosporidiosis cases reported to the MIS in the Aragón region, focusing on the territorial, temporal, and sociodemographic distribution within the paediatric population of the Autonomous Community.

## 2. Materials and Methods

**Figure 1:**
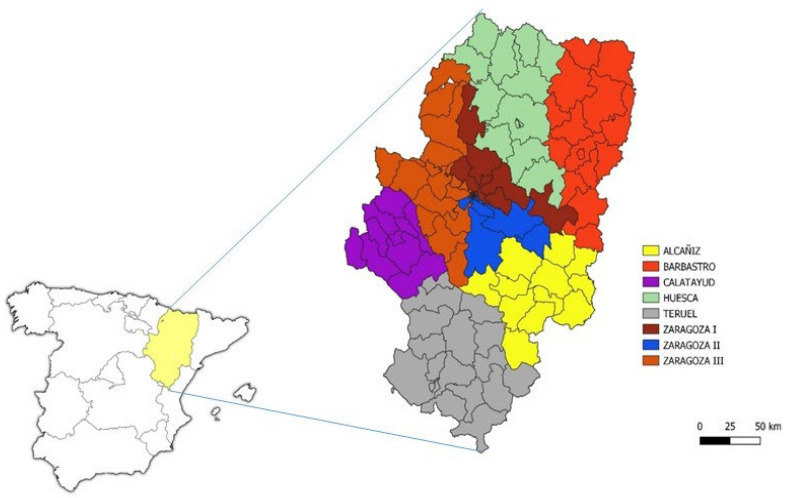
Representation of the healthcare map of Aragón (Spain), divided into 8 sectors within the Autonomous Community (Alcañiz, Barbastro, Calatayud, Huesca, Teruel, Zaragoza I, Zaragoza II and Zaragoza III).

This study was conducted in the Autonomous Community of Aragón, located in northeastern Spain, characterised by extreme climates in both summer and winter, and low rainfall. The region lies between the Pyrenees and Iberian mountain systems, with the Ebro River valley running through it (Figure 1) [28]. Aragón consists of three provinces: Zaragoza (the autonomous capital), where the majority of the population resides (979,365 inhabitants in 2023); Huesca (226,046 inhabitants in 2023); and Teruel (135,045 inhabitants in 2023). The population of Aragón is ageing and largely concentrated in the provincial capitals [28].

This is a retrospective descriptive study of proportions in which notification and registration data related to giardiasis and cryptosporidiosis in the healthcare system were analysed, from 1 January 2012 to 31 December 2021.

The data sources used for the study were as follows:

The number of updated health cards per year was obtained through the Aragonese Institute of Statistics (AIS). These cards reflect annual paediatric healthcare coverage [28].

The data corresponding to reported cases in the studied region were provided by the MIS, managed by the Department of Epidemiological Surveillance of the Directorate General of Public Health of the Government of Aragón. These data correspond to children aged 0–14 years in the Autonomous Community of Aragón, distributed across different health sectors of the region from 2012 to 2021, and stratified by age group [29]. A retrospective analysis was conducted between 2012 and 2021, as the Microbiological Information System was progressively implemented in Aragón (Spain) between 2012 and 2018. The study period was extended to assess the impact of the SARS-CoV-2 pandemic on case reporting to the system. Data from samples taken on the same day from the same patient were excluded to eliminate duplications. Nevertheless, to maintain confidentiality, the data were provided anonymously, which may result in a small proportion of duplicate cases reported to the system. These duplicates are assumed to be methodological errors. Data on climatology and rainfall in the years of study were obtained from the Aragonese Open Data on climate [30].

The studied variables related to data were gender (male/female), age (categorised according to MIS: 1–11 months; 12–23 months; 2–4 years; 5–14 years), healthcare sector (sectors of Zaragoza city, denoted as I, II, and III; Calatayud (Zaragoza), Huesca, Barbastro (Huesca), Teruel, and Alcañiz (Teruel), season (spring, summer, autumn, winter) and weather (average monthly temperature and annual precipitation in millimetres of rainfall (mm), equivalent to 1 L per m^2^). The location of the healthcare sectors is shown in Figure 1.

The collected data were gathered into a database and analysed using the IBM^®^ SPSS^®^ Statistics v.26 software (University of Zaragoza License). The results of the statistical analysis of the variables are expressed in percentages, proportions and infectivity rates.

The incidence rate (per 10,000) for *Giardia lamblia* and *Cryptosporidium* spp. was calculated annually and by health sector. The proportion of *Giardia* and *Cryptosporidium* infection cases was estimated through frequencies (N) and percentages (%) of total cases by Health Sectors. The incidence rate (IR) of both protozoa infections (giardiasis and cryptosporidiosis) was calculated in relation to the number of reported cases in the Microbiological Information System (MIS) and the number of health cards of the paediatric population by health sector and by year. To calculate this, the number of cases of *giardiasis* or *cryptosporidiosis* in a specific year (2012–2021) was used as the numerator and divided by the estimated population as of December 31 of the corresponding year (denominator), following the recommendations provided in the literature [31]. The comparison of the values of a quantitative variable against those of a dichotomous variable was performed using Student’s *t*-tests or Mann–Whitney U tests, depending on the result of the normality test. The relationship between two qualitative variables was assessed using the Chi-square (χ^2^) statistical test or Fisher’s exact test in the case of sparsely populated cells. To determine which modalities of the variables had a significant association, the standardised Haberman residuals were analysed.

The rates were expressed exponentially (10^−4^) to facilitate comparison of the results.

Ethical considerations: The study was approved by the organisations that provided the data (Public Health Directorate, Government of Aragón) and by the Research Ethics Committee of the Autonomous Community of Aragón (CEICA) (PI 16/0235).

## 3. Results

During the study period, a total of 1424 cases of parasitic diseases in children aged 0 to 14 years, caused by the two protozoa under investigation, were reported. Of these, 1056 were due to *Giardia* and 368 were attributed to *Cryptosporidium*. The distribution by province and sector of the total cases in children of *Giardia* and *Cryptosporidium* recorded from 2012 to 2021 is shown in Table 1.

The cases are presented based on the distribution across the reference hospitals where the cases were reported. Table 2 shows the infectivity rates for giardiasis and cryptosporidiosis, respectively, by year and healthcare sector. It is important to note the high number of reported cases of giardiasis and cryptosporidiosis in 2012, with 172 and 91 cases, respectively, and in 2018, with 167 and 131 cases, respectively, which also resulted in the highest infectivity rates.

The proportion of giardiasis was significantly higher in sectors II and III of Zaragoza (*p* < 0.001), while the proportion of cryptosporidiosis was higher in Calatayud, followed by sector II of Zaragoza and sector III of Zaragoza (*p* < 0.001). Table 2 shows the infection rates by health sector and year for both parasitic diseases. The highest infection rate for *Giardia* was recorded in Teruel in 2018 (36.1 × 10^−^⁴), followed by the Zaragoza Sector III healthcare sector in 2012 (17 × 10^−^⁴), and Zaragoza Sector II in 2013 (15.7 × 10^−^⁴). The downward trend in cases from 2012 is notable, with occasional slight increases.

In the case of *Cryptosporidium* spp. (Table 2), the locations with the highest incidence rates were Calatayud in 2012 (25.1 × 10^−4^) and in 2015 (13.2 × 10^−4^), followed by Sector Zaragoza III in 2012 (12.2 × 10^−4^). It is worth noting that very few cases were recorded in some sectors, such as Alcañiz and Teruel (Table 2).

**Figure 2:**
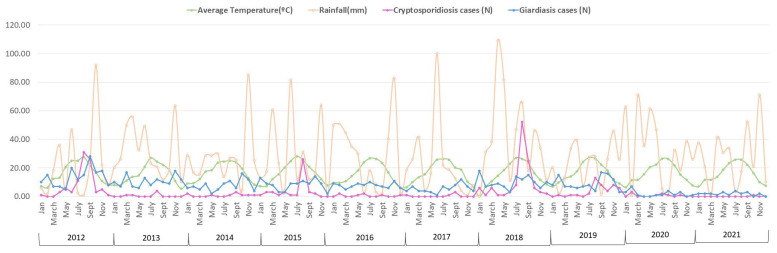
Temperatures, rainfall, giardiasis and cryptosporidiosis cases in children in Aragón (Spain) (2012–2021). The total number of cases of both parasitoses is shown for each month, along with the average temperature and rainfall for the corresponding period. Peaks in cryptosporidiosis cases are observed shortly after periods of maximum rainfall, as well as the seasonal trends of both diseases and the appearance of cryptosporidiosis case surges approximately every three years.

When examining the association between sex and parasitic infection during the study period, boys were found to be significantly more likely than girls to be infected with *Giardia* (4.9% vs. 3.8%) (*p* < 0.001). The proportion of boys and girls infected with *Cryptosporidium* was almost identical (1.4% vs. 1.3%) (Table 3). In addition, the age group with the highest proportion of giardiasis cases was the 5–14 years age group (median: 5, IQR: 6), while for cryptosporidiosis, the most predominant age group was 2 to 4 years (median: 2, IQR: 1). Upon analysing the seasonal distribution, the highest number of cases was observed in autumn for *Giardia lamblia* and in summer for *Cryptosporidium* spp. (Figure 2).

In Table 4, the cases of *Giardia* and *Cryptosporidium* are compared with the annual averages of temperature and rainfall (2012–2021 series). Significant differences were found between the notification of *Giardia* and *Cryptosporidium* regarding temperature, but not in relation to rainfall (Table 4).

By province, the high number of giardiasis cases in Teruel in 2018 stands out, particularly during July and August. In August, when the highest number of cases (12) was recorded, it coincided with heavier rainfall (73.2 mm). However, despite continued rainfall in September and October (72 mm and 138.6 mm, respectively), no further cases were reported.

The data analysis by month and province was limited to Zaragoza due to a low number of cases in other provinces, which were also scattered throughout the year. In Zaragoza, however, cases of giardiasis begin to increase in August and reach their highest levels during the autumn months. This trend suggests a seasonal pattern, with a notable rise starting in late summer and peaking in the fall.

Conversely, reported cases of cryptosporidiosis have shown a significant spike every three years—in 2012, 2015, and 2018—particularly during August and September following episodes of heavy rain. Interestingly, while heavy rains also occurred in other years, such as 2017 and 2020, these did not coincide with a similar increase in cases (Figure 2). This suggests that additional factors beyond rainfall may influence the outbreak pattern, or that specific conditions during the peak years amplified transmission in ways not replicated in the other rainy years.

## 4. Discussion

The epidemiological study conducted on cases of *Giardia* and *Cryptosporidium* infections holds significant relevance as it allows for the observation of the evolution of these infections over a 10-year period. The results, calculated through infectivity rates, highlight specific geographical areas with higher incidence, providing a solid basis for further investigation into the causes of these elevated rates. Moreover, the analysis of health registry systems reveals important deficiencies and identifies opportunities for improvement to ensure these records are more complete and useful. The persistence of cases of giardiasis and cryptosporidiosis in the paediatric population underscores the need for healthcare professionals to remain vigilant for symptoms compatible with these infections, contributing to more timely diagnosis and treatment. Additionally, the data obtained highlight the urgency of implementing informational campaigns on hygienic measures to prevent the spread of these parasites, alongside efforts by public health authorities to ensure the proper maintenance of water resources in contact with the population. These actions are essential not only for controlling the transmission of these pathogens in the general population but also for protecting vulnerable individuals whose infections could lead to severe consequences. *Giardia lamblia* and *Cryptosporidium* spp. are two protozoan pathogens frequently identified as causes of gastroenteritis in the studied area, particularly affecting children.

Since both giardiasis and cryptosporidiosis are notifiable diseases, reported cases from 2012 to 2021 were studied. Some difficulties were encountered in data collection and analysis due to the progressive incorporation of hospital laboratories from various health sectors into the microbiological information system. The laboratory from the Huesca sector was integrated in 2015, those corresponding to Teruel and Barbastro in 2017, and the laboratory from Zaragoza II began analysing cases from the Zaragoza I sector in 2015 and, therefore, has included these data in the Microbiological Information System since then. Among the limitations, in addition to the progressive implementation of laboratories into the system, we also found the availability of paediatric health cards, which are divided by sex but not by age groups, making it difficult to estimate the proportion relationship for this variable. However, it was possible to establish a statistical relationship between variables such as sex and age.

The results of this study show that the highest number of cases for both *Giardia* and *Cryptosporidium* corresponds to the Zaragoza sectors. The number of reported cases reflects the demographic distribution of the region, with a large portion of the population concentrated in the capital, Zaragoza (682,513 inhabitants in 2024), in contrast to the other provincial capitals in the region—Huesca, with 55,217 inhabitants, and Teruel, with 36,155, the latter being the least populated provincial capital in Spain [28].

There is no relationship between the variation in rates across the health sectors studied for either *Giardia* or *Cryptosporidium*, suggesting a local and independent pattern of dissemination. However, population movements, especially during holiday periods, should be considered, as they may facilitate the spread of these diseases [25,32,33]. It would be necessary to identify the genotypes and populations being disseminated to rule out any relationship between the protozoa found in different populations.

The results reflect a reduction in cases in 2020, a year when contact between people was limited due to the spread of SARS-CoV-2, along with a reduction in performance, and even modifications in the techniques used in confirmatory analyses [34]. This decrease in 2020 is observed across all health sectors studied. However, in 2021, an upward trend reappears, with a notably high rate in Teruel (10.2 × 10^−4^). This increase suggests the ongoing presence of reservoirs, potentially in the form of chronic, asymptomatic carriers or environmental sources that facilitate continued transmission.

Regardless of these variations, Teruel’s rate of giardiasis has been the highest in Aragón every year. Unfortunately, there are no official data prior to 2018 to determine if this trend is consistent. Only the study by Ramos et al. [35], published in 2004, reported an incidence of giardiasis of 20% in children in Teruel, a much higher proportion than in the rest of Spain. These data suggest that giardiasis may be a long-standing issue in the health area of Teruel, as confirmed by Labay [36], who links cases of giardiasis in the same territory to related symptoms in the paediatric population.

A 2021 study conducted in Castellón (Valencian Community, Spain, neighbouring the province of Teruel) reported incidence rates of giardiasis ranging between 0.2 × 10^−5^ and 1.6 × 10^−4^ [37], substantially lower than those observed in Teruel, where rates spiked to a maximum of 36.1 × 10^−4^ and a minimum of 4.07 × 10^−4^. At the European level, research from Scotland estimated an average incidence rate of 5.4 × 10^−4^ among children over an 8-year period (2011–2018). These comparisons suggest a notably higher incidence in certain regions of Spain, potentially indicating endemicity of these protozoan infections in specific areas, particularly in Teruel, which stands out with its unusually high rates [38].

According to international standards, the incidence of giardiasis in Aragón significantly surpasses the acceptable annual infection risk for the general population [39]. The U.S. Environmental Protection Agency (EPA) recommends that this risk should not exceed 10^−4^, or 1 infection per 10,000 inhabitants [39]. However, for the paediatric population, the EPA’s risk models estimate an annual *Giardia lamblia* infection risk of up to 20 × 10^−4^, with potential peaks as high as 250 × 10^−4^ [39]. These figures highlight the heightened vulnerability of children and indicate that giardiasis in Aragón exceeds tolerable risk levels, emphasising a need for strengthened public health measures to reduce infection rates.

In the case of *Cryptosporidium*, incidence rates are much lower than those of giardiasis, with the Calatayud health sector having the highest *Cryptosporidium* spp. infectivity rate for several years. It is striking that significant increases were recorded in 2012, 2015 and 2018 across all sectors of Zaragoza and Huesca, while in Teruel and Alcañiz, there are hardly any cases.

Interestingly, an outbreak of cryptosporidiosis occurred in 2023 in several towns in Zaragoza, suggesting a cyclical increase in cases. During 2023, Spain experienced an unusual increase in cases of cryptosporidiosis, surpassing 3400 reported cases by 31 October [29]. This rise was mainly linked to exposure to swimming pools and recreational waters, as well as outbreaks associated with the consumption of tap water, some of which were of significant magnitude. Molecular studies revealed that many of these infections were caused by genetic variants uncommon in Spain [40].

This phenomenon is not exclusive to Spain; other European countries have reported similar increases. Among the contributing factors are the extreme weather conditions that marked the summer of 2023, which, becoming increasingly frequent, heighten the risk of waterborne diseases. These situations pose challenges for water treatment plants, especially in areas lacking advanced facilities, such as small towns or rural regions. In these locations, where water is disinfected only with chlorine-based derivatives, *Cryptosporidium* spp. oocysts are not eliminated, increasing vulnerability.

In terms of impact, cryptosporidiosis is generally a self-limiting disease in healthy children and adults, but it poses a significant risk for immunocompromised individuals, who may develop severe infections. This highlights the need to strengthen epidemiological and genotypic surveillance and to implement environmental protocols to monitor the presence of *Cryptosporidium* spp. in water and assess the effect of weather events on water treatment plants [40].

Additionally, preventing outbreaks associated with swimming pools and recreational waters requires proper maintenance and adherence to hygiene measures by the population. These efforts, combined with enhanced monitoring, are essential to address the growing impact of extreme weather events on the transmission of waterborne diseases [40]. According to the report by the Spanish Ministry of Health, seven outbreaks of cryptosporidiosis were reported in 2018, primarily linked to household and waterborne transmission [21]. This transmission pattern is also confirmed at the European level between 2012 and 2014, according to a study by Cacciò [41]. Previous incidence rates (2001–2005) in countries such as Germany, the Netherlands, and England were 1.29, 1.93, and 8.25 × 10^−5^, respectively, with these countries reporting the highest number of outbreaks [41].

In an attempt to relate the results obtained to climatological events, it should be noted that during 2012, according to the State Meteorological Agency (SMA) [42], there was an increase in temperatures in Spain, and according to the Basic Climate Information System of Aragón, the summer was hotter and drier than usual, with a rainier autumn than other years. These situations of intense rainfall can cause damage to water purification infrastructures that favour the presence of (oo)cysts in drinking water and, therefore, the increase in the number of cases and even the appearance of outbreaks [43,44]. These factors were repeated in both 2015 and 2018, where the highest incidence rate of *Giardia*, in Teruel, coincided with the maximum number of millimetres of rainfall in the province [30].

As with giardiasis, there was a significant reduction in the number of cryptosporidiosis cases during 2019 and 2020, coinciding with the COVID-19 pandemic and related confinement measures, which greatly reduced interpersonal contact [34]. As mentioned previously, this suggests a significant role for person-to-person transmission. However, it also coincided with a decrease in doctor visits and an increase in the use of rapid immunochromatography tests for diagnosis, replacing microscopic visualisation, which has lower specificity for detecting *Cryptosporidium* compared to *Giardia* [14,45]. According to other authors [43,44], person-to-person transmission plays an important role as a transmission mechanism, especially in the case of children. However, the consumption of contaminated water and raw food irrigated with contaminated water are the main transmission routes for these protozoa. In developed countries, there are still populations where water purification consists only of disinfection by adding chlorine, which is not effective against *Giardia* and *Cryptosporidium* (oo)cysts. However, the rapid dissemination of both parasites often coincides with infrastructure problems that lead to accidental contamination of water near collection points. Climatic phenomena that have occurred in the years of increasing cases may have been decisive, in damaging the infrastructures. Although it is very difficult to prevent inclement weather, the recommendations of the Health Alerts and Emergencies Coordination Centre [40] include environmental monitoring protocols to assess the water risk of all supplies. The presence of hypertransmissible genotypes also makes transmission more effective.

Although there are no differences by sex and age, as the distribution is similar, it can be observed that *Cryptosporidium* infection is more frequent in preschool-aged children (2–4 years), while *Giardia* is more frequent at school-aged children (5–14 years), following the trend already described by other authors, at national level in the Basque Country [11], or at internationally, such as in Eastern European countries [46]. In the study by Ramos [35], however, reported cases of *Giardia* occurring earlier in Teruel (0–4 years). Nevertheless, it is clear that person-to-person transmission within schools is frequent, ranging from non-compulsory nursery school to primary school classrooms.

Seasonally, *Giardia* cases were highest in autumn, while *Cryptosporidium* cases peaked in summer during the studied years. The same trend has been observed in other countries as reported by international studies [47], as well as that of Saura-Carretero in Spain [37]. The hypothesis that may explain this trend is that increased transmission during the summer months, due to the use of recreational water and other outdoor activities common among children, favours transmission. However, detection may occur more easily when asymptomatic children return to school, leading to person-to-person transmission [48,49].

To control the transmission of *Giardia* and *Cryptosporidium*, preventive measures must focus on several key areas. Firstly, regarding person-to-person transmission, it is essential to promote hygiene practices such as frequent handwashing, particularly after using the restroom or before preparing food [50]. In terms of hygienic measures, regular cleaning of surfaces and proper food handling should be encouraged. Concerning water treatment, it is crucial to ensure that drinking water is adequately filtered and disinfected using effective technologies such as ultraviolet radiation or ozonation [44], as these protozoa are resistant to chlorine. Additionally, recreational water, such as in swimming pools and lakes, must be treated with methods that eliminate pathogens, preventing faecal contamination [51]. Finally, in schools, it is important to implement educational programs and maintain clean environments, emphasising hand hygiene and proper cleaning of toys and common areas to prevent the spread of these infections among children [27].

## 5. Conclusions

This study has allowed for the analysis of the proportion of giardiasis and cryptosporidiosis cases reported to the MIS in the Aragón region, focusing on the territorial, temporal and sociodemographic distribution within the paediatric population of the Autonomous Community. The results provide a detailed view of the prevalence of these infections across different areas and time periods, as well as the associated sociodemographic and climatological characteristics, contributing to a better identification of areas and characteristics that require attention and specific preventive measures. In the case of *Giardia*, a higher proportion of cases occurs in boys aged 5–14 years, particularly towards the end of August. Reported cases remain relatively constant over time, although the presence of asymptomatic individuals and reservoirs needs to be investigated, especially in areas such as the province of Teruel. However, *Cryptosporidium* affects boys and girls aged 2–4 years equally, with higher frequency in summer. A notable increase in cases occurs every three years, which can be partly attributed to climatic phenomena, although the interplay of other factors must be further investigated. All the results highlight the importance of reporting and studying cases for effective control of the transmission of both protozoa and the prevention of outbreaks. This study provides the foundation for future research on the evolution of giardiasis and cryptosporidiosis cases in children in Spain.

## Figures and Tables

**Table 1:** Total number of reported cases of giardiasis and cryptosporidiosis in the paediatric population within the healthcare sectors of Aragón (Spain) from 2012 to 2021. The average number of health cards for each sector during the study period, the total cases of giardiasis and cryptosporidiosis (both combined and for each parasite individually), and the percentage of infected children in each case are presented.

Province	Health Sector	Average Number of Health Cards for Children	Giardiasis No. of Cases (%)	*p*	Cryptosporidiosis No. of Cases (%)	*p*	Total No. of Cases (%)
**ZARAGOZA**	ZGZ I + II	44,116	525 (1.2%)	0.001	170 (0.4%)	0.001	695 (1.6%)
	ZGZ III	48,063	364 (0.75%)		128 (0.27%)		492 (1.12%)
	Calatayud	5789	27 (0.46%)		34 (0.58%)		61 (1.15%)
	**Total Zaragoza**	**35,521**	**916 (2.58%)**		**332 (0.93%)**		**1248 (3.5%)**
**HUESCA**	Huesca	15,549	23 (0.15%)		23 (0.15%)		46 (0.32%)
	Barbastro	15,784	3 (0.02%)		10 (0.06%)		13 (0.09%)
	**Total Huesca**	**15,667**	**26 (0.17%)**		**33 (0.21%)**		**59 (3.8%)**
**TERUEL**	Alcañiz	10,292	55 (0.59%)		3 (0.03%)		58 (0.62%)
	Teruel	10,145	59 (0.64%)		0		59 (0.64%)
	**Total Teruel**	**10,218**	**114 (0.61%)**		**3 (0.01%)**		**117 (1.1%)**
**Total Aragón**		**24,231**	**1056 (4.3%)**		**368 (1.5%)**		**1424 (5.8%)**

The % has been calculated with respect to the number of health cards in each population.

**Table 2:** Infectivity rates for giardiasis and cryptosporidiosis, respectively, by year and healthcare sector in Aragón (Spain), based on reported cases in the paediatric population from 2012 to 2021 to the Microbiological Information System (MIS).

	Giardiasis							Cryptosporidiosis						
	(Rate ×10^−4^)							(Rate ×10^−4^)						
YEAR	HEALTH SECTOR							HEALTH SECTOR						
	ZGZ	ZGZ III	CLY	HUESCA	BARBASTRO	TERUEL	ALCAÑIZ	ZGZ	ZGZ III	CLY	HUESCA	BARBASTRO	TERUEL	ALCAÑIZ
	I + II *							I + II *						
2012	14.5	17	12.5	ND	ND	ND	3.6	3.2	12.2	25.1	ND	ND	ND	1
2013	15.7	9.8	3.1	ND	ND	ND	7.6	0.8	0	3.1	ND	ND	ND	0
2014	10.5	7.1	4.8	ND	ND	ND	4.7	0.6	1.2	1.6	ND	ND	ND	0
2015	6.7	7.1	9.9	3.1	ND	ND	3.8	1.0	5.6	13.2	0	ND	ND	0
2016	6.2	7.7	5.1	1.2	ND	ND	5.8	0.0	0.8	6.8	0	ND	ND	0
2017	5.1	4.3	0	5.1	0.6	ND	8.8	0.2	0.8	1.7	1.9	1.2	ND	0.9
2018	8.9	7.5	3.5	2.5	0.6	36.1	6.9	10.7	3.2	3.5	6.4	4.4	0	0
2019	7.4	8.8	1.8	1.3	0	3.1	5.9	4	1.6	0	5.1	0	0	0
2020	1.1	8.6	0	1.3	0	4.1	0	0.3	1	0	0.6	0	0	1
2021	0	4.1	3.8	0	0.6	10.2	0	0	0.2	0	0.6	0.6	0	0

* The population rates of Sector I have been combined with those of Sector II since 2015. Prior to 2015, their cases were not automatically reported to the MIS. ZGZ: Zaragoza; CLY: Calatayud. Heatmap color: Red: Max rate (37-20); Dark Orange: Mid-High rate (19.9-15); Light Orange: Medium rate (14.99-5); Yellow: Mid- Low rate (4.99-1.5); Light Green: Low rate (1.49-0.1); Dark Green: Min. rate (0).

**Table 3:** Statistical analysis of the proportions of Giardia and Cryptosporidium cases (2012–2021) by sex.

Variable N(%)		*Giardia lamblia*		*Cryptosporidium* spp.	
		Cases	*p*	Cases	*p*
Sex	Boy	604 (4.9)	0.001	197 (1.6)	0.373
	Girl	452 (3.8)		171 (1.4)	

**Table 4:** Statistical analysis of the climatology in Aragón (Spain) (rainfall and temperatures) in relation to cases of giardiasis and cryptosporidiosis in the child population (2012–2021).

Protozoan	Rainfall (mm)				Temperature (°C)			
	Mean (SD)	95% CI (Lower; Upper)	Median (IQR)	*p*	Mean (SD)	95% CI (Lower; Upper)	Median (IQR)	*p*
***Giardia lamblia*** (N = 1034)	30.40 (25.06)	28.80; 31.90	23.50 (34.1)	0.442	16.74 (6.96)	16.35; 17.21	16.90 (12.80)	<0.001
***Cryptosporidium* spp.** (N = 365)	31.90 (26.58)	29.10; 34.60	26.40 (34.35)		21.21 (6–38)	20.54; 21.87	23.60 (9.70)	

## Data Availability

Databases from the Health Department of Aragón were anonymised and private, based on public data from MIS (https://www.mscbs.gob.es/estadEstudios/estadisticas/estadisticas/estMinisterio/microbiologica.htm accessed on 7 December 2024).

## Abbreviations

The following abbreviations are used in this manuscript:

MDPI	Multidisciplinary Digital Publishing Institute
DOAJ	Directory of open-access journals
TLA	Three-letter acronym
LD	Linear dichroism
